# *Toxorhynchites* Species: A Review of Current Knowledge

**DOI:** 10.3390/insects11110747

**Published:** 2020-10-30

**Authors:** Claire L. Donald, Padet Siriyasatien, Alain Kohl

**Affiliations:** 1 Institute of Molecular, Cell and Systems Biology, College of Medical, Veterinary and Life Sciences, University of Glasgow, Glasgow G12 8QQ, Scotland, UK; 2 MRC-University of Glasgow Centre for Virus Research, Glasgow G61 1QH, Scotland, UK; alain.kohl@glasgow.ac.uk; 3 Vector Biology and Vector Borne Disease Research Unit, Department of Parasitology, Faculty of Medicine, Chulalongkorn University, Bangkok 10330, Thailand; padet.s@chula.ac.th

**Keywords:** *Toxorhynchites*, elephant mosquito, biological vector control

## Abstract

**Simple Summary:**

Mosquitoes are well known to spread diseases when they take a blood meal. However, not all species feed on blood but instead get their nourishment from other sources. One such species is *Toxorhynchites*, which are a paradox among mosquitoes. These mosquitoes are entirely non-blood feeding and, as a result, are not considered to be harmful to human health. Indeed, since their larvae feed on the larvae of pest species and other aquatic insects, they are a potential counter measure against the spread of mosquito-transmitted diseases. Their effective application has been hampered due to a lack of understanding and inconsistencies in their descriptions. This review aims to build upon previously published information and summarize recent findings to support their use in combating mosquito-transmitted infections.

**Abstract:**

The increasing global incidence of mosquito-borne infections is driving a need for effective control methods. Vector populations have expanded their geographical ranges, while increasing resistance to chemical insecticides and a lack of effective treatments or vaccines has meant that the development of vector control methods is essential in the fight against mosquito-transmitted diseases. This review will focus on *Toxorhynchites*, a non-hematophagous mosquito genus which is a natural predator of vector species and may be exploited as a biological control agent. Their effectiveness in this role has been strongly debated for many years and early trials have been marred by misinformation and incomplete descriptions. Here, we draw together current knowledge of the general biology of *Toxorhynchites* and discuss how this updated information will benefit their role in an integrated vector management program.

## 1. Introduction

Mosquitoes are responsible for the transmission of numerous pathogens which cause significant mortality and morbidity in both humans and animals, as well as substantial economic losses in many parts of the world. Rising global temperatures coupled with increasing travel and trade have led to the expansion of the geographical range of a number of important vector species. This in turn has contributed to the emergence or reemergence of mosquito-borne pathogens in new areas and communities. To date, a large number of mosquito-borne infections lack effective vaccines or specific antiviral therapies, and so vector control strategies play a vital role in their regulation. Many of these control methods are heavily reliant on the use of insecticides. Resistance to the four commonly used classes of insecticides—pyrethroids, carbamates, organophosphates and organochlorines—is now known to be extensively widespread and has facilitated a rise in the occurrence of these diseases [1,2,3,4]. In addition, concerns over environmental damage and negative effects on non-target organisms, not to mention sustainability and cost issues, have led to a drive to develop novel vector control strategies to prevent a substantial increase in the incidence of infections [5,6,7,8].

Biological control methods which directly target the mosquito therefore represent an important alternative for the reduction or elimination of mosquito-borne diseases [9]. One promising method is the employment of natural enemies which feed on the mosquitoes’ aquatic life stages. This includes mosquitoes within the genus *Toxorhynchites* which may prove to be a useful tool. Unfortunately, limited studies have been performed to date, which have provided insufficient evidence for us to fully benefit from their use. Expanding our knowledge of their behavior, as well as their general biology, will not only aid their practical application as a biological control agent but can also be used to improve our understanding of the biology of blood feeding species. This article serves to build upon what has been previously described on *Toxorhynchites* [10,11,12] by expanding on recent updates of our current understanding of their biology and potential role in controlling mosquito-borne infections.

## 2. General Biology

*Toxorhynchites* are commonly referred to as ‘elephant mosquitoes’, or ‘mosquito eater’, partly due to their size but also because of their trunk-like proboscis, which is large and curves downward to a point to aid consuming nectar [12]. This distinct shape is adapted to feeding on sugar and does not allow the females to consume a bloodmeal [13]. Both sexes are phytophagous and feed exclusively on nectar and other sugary substances. As a result, they are not involved in the transmission of pathogens to humans or animals and are therefore not considered to be of medical importance. Instead, *Toxorhynchites* larvae prey on other mosquito larvae, notably *Aedes* species which spread high-profile public health pathogens, such as Zika (ZIKV), dengue (DENV), chikungunya (CHIKV) and yellow fever (YFV) viruses [14]. In stark contrast to the behavior of the placid adults, the larvae are voracious predators and are described as consuming large quantities of living and non-living prey, allowing them to acquire all the necessary proteins and fats for successful oogenesis in adulthood [15]. *Toxorhynchites* are not the sole example of non-blood feeding mosquitoes. *Malaya* and *Topomyia* are also nectivorous and as a result do not exhibit host-seeking behavior [16]. However, due to their feeding preferences, neither have any benefits as biological control agents. *Toxorhynchitine* species are active during the day and are largely found in the tropics, although a few species are present in Asia, North America, Fiji and the Samoan Islands [11]. The majority of species are forest-dwelling, although *Tx. splendens* has been shown to inhabit coastal regions [17].

## 3. Taxonomic Classification

*Toxorhynchites* is the only genus within the Toxorhynchitini tribe and consists of approximately 90 species across four subgenera: *Toxorhynchites* (51 sp.), *Lynchiella* (16 sp.), *Afrorhynchus* (19 sp.) and *Ankylorhynchus* (4 sp.) [10,18,19,20,21,22,23,24,25,26,27]. Although *Toxorhynchites* are restricted to the Old World, *Lynchiella* and *Ankylorhynchus* are present in the New World, while *Afrorhynchus* are found in Africa. Members of the genus are morphologically similar to each other despite their wide geographical distribution, making identification at the species level very difficult [28]. This, combined with a lack of taxonomic information [11], has led to some species being described on more than one occasion under different names. Misidentification of species has had significant consequences on their success as a biological control tool (discussed in Section 8). Furthermore, the phylogenic relationships of *Toxorhynchites* have not been fully determined [20,29,30,31]. Although it was initially suggested that *Toxorhynchites* is an independent subfamily within *Culicidae* [32], subsequent reports placed it somewhere within the *Culicinae* subfamily [29,33,34,35,36,37,38,39,40]. It would be interesting to understand if hematophagy was lost in *Toxorhynchites* or was an adaptation that arose in specific lineages after they diverged. As blood feeding is believed to have been independently acquired and lost on several occasions in dipterans, it is not straightforward to suggest the correct order of events with presently available information [41]. Establishing the key genes involved in the evolution of a non-biting lifestyle could provide an alternative approach to mitigating disease transmission.

## 4. Development

### 4.1. Eggs

**Figure 1:**
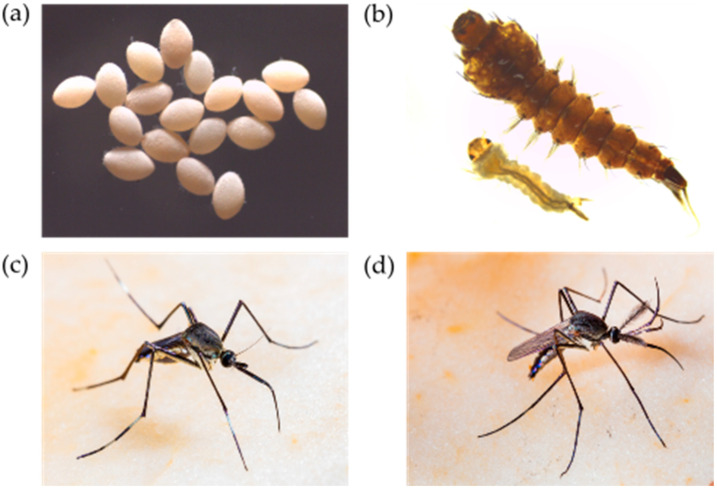
*Toxorhynchites splendens* life stages. (**a**) Eggs; (**b**) comparison between fourth instar larva of *Tx. splendens* and *Cx quinquefasciatus*; adult (**c**) female and (**d**) male.

*Toxorhynchites* females are autogenous and acquire all the protein required for oogenesis and vitellogenesis during their larval stages. Their follicles (oocytes) are continually produced and develop at separate times, which allows the female to be able to oviposit as soon as she discovers a suitable ovipositing site [42,43]. All species produce eggs that are similar in appearance (Figure 1a) [19]. These eggs are bright-white or yellowish, oval-shaped [10,44] and are laid on the surface of water in both natural and artificial containers, including tree cavities or man-made water containers, such as disused tires or flowerpots [43,45]. In this way, *Toxorhynchites* are not searching directly for prey but rather a suitable ovipositing site. Virtually any container which is able to hold water will make a suitable habitat, provided that it is partly shaded. Importantly, these oviposition sites are also used by other mosquito species, including disease vectors [46,47,48,49]. The female lays her eggs individually mid-flight into small batches and they will incubate for 40–60 h depending on relative temperature and humidity. The eggs have a water-resistant coating surrounding a hydrophilic interior and must maintain contact with water as they cannot endure desiccation. Females must distribute their eggs widely in order to minimalize the risk of cannibalism amongst her offspring and ensure that adequate prey is available [50,51]. While this natural behavior is beneficial for targeting a greater number of vector breeding sites, cannibalism is a considerable cause of mortality in some *Toxorhynchites* species and is a key factor to be considered when exploiting them as a biological control tool [10,52,53].

### 4.2. Larvae

The instars of all *Toxorhynchites* species are voracious predators and will prey on any larvae or other similarly sized aquatic organisms sharing the same water source (Figure 1b). Primarily, this is thought to consist of the larvae of other mosquito species, as well as Chironomidae and Tipulidae larvae, dragonfly nymphs and aquatic worms [54,55,56]. However, they will also feed on the larvae and pupae of their own species, particularly when other food supplies are few or absent [11,57]. Larval feeding rates vary depending on a variety of biotic (prey size and abundance) and abiotic (water temperature and sunlight levels) factors; however, during their maturation, a single larva may consume up to 5000 prey larvae [10,12]. *Toxorhynchites* larvae are opportunistic hunters and do not search for their prey. Instead, they use mechanoreceptors to detect the presence of their prey. Once within range, the larvae will actively swim towards it and seize it in their powerful mandibles before consuming it [58,59]. Their mandibles have comb-like extensions which they use to grip onto prey. Although early instar larvae require live food to stimulate a feeding response, fourth instar larvae will also feed on immobile detritus. Incidences of prepupal killing when fourth instar larvae on the edge of pupation kill prey but do not eat it have also been documented (reviewed in [11]). It is hypothesized that this decreases the number of potential predators in their vicinity prior to transitioning into the vulnerable pupal stage [60]. Other non-lethal affects have also been observed. For example, the presence of predatory larvae during early developmental stages of *Ae. aegypti* larvae resulted in decreased development rates, shortened adult lifespans [61] and the production of fewer eggs [62]. The presence of predators in oviposition sites has been shown to have species-specific behavioral effects. While *Aedes* species were observed to be unaffected, *Culex* species showed strong behavioral avoidance of sites containing predators [63,64,65]. In addition, studies by Juliano and colleagues have noted that *Ae. triseriatus* have developed adaptations to protect themselves from predation by minimizing their movements and decreasing their foraging behavior in the presence of *Tx. rutilus* [66]. Fully understanding these effects on population dynamics will be important for their implementation as a biological control strategy.

Larval development is dependent on a variety of variables, such as temperature, light and prey density (reviewed in [10]). Decreases in these factors result in lengthened larval development, which allows fourth instar larvae to overwinter until more favorable conditions arise. Developmental timelines for each life stage have been described elsewhere [10,11]. Further to this, recent research has shown that *Toxorhynchites* larvae need a living gut microbiota to develop past the first instar stage and that these bacterial communities are consistent to those of their prey [67]. Larvae that fail to acquire these microbes exhibit developmental defects consistent with nutritional deficiencies.

### 4.3. Adults

Adults are colorful and exceptionally large with a wingspan of approximately 12 mm in size (Figure 1c,d) [59]. Their bodies are covered in iridescent scales of various colors with tufts of colored setae on their abdomens. With the exception of oviposition behavior [68,69,70,71,72,73], very little has been done to understand the biology of *Toxorhynchites* species adults in the wild. Oviposition behavior has been observed in a number of species (reviewed in [10]). The female lays her eggs following the completion of a distinctive flight pattern consisting of a series of vertical, oval loops that decrease in size as she nears the oviposition site. The eggs are laid individually, and the process is either repeated or the female departs. As they do not need to land in order to oviposit, *Toxorhynchites* are able to lay eggs in containers with obstructed openings which do not allow easy access to the surface of the water. It is also likely that aerial oviposition will reduce the likelihood of predation [43]. 

It is known that the pupal stage generally lasts between 3–7 days, following which the adults emerge during the day. However, it varies depending on species, whether this is protogynous (females emerge before males) [74] or protandrous (males emerge before females) [75]. Little is known about nectar source preference, such as flower species, color or shape, or whether the adults play a role in pollination. A study by Godoy and colleagues investigated the morphology of the midgut of *Tx. theobaldi.* They demonstrated that in both sexes, the midgut strongly resembled that of male hematophagous mosquito species and was adapted to high sugar diets [76]. Similarly, the salivary glands of both sexes of *Tx. splendens* are morphologically and biochemically similar, which is unlike those of hematophagous species that display sexual dimorphisms [77]. Their salivary glands also appear morphologically distinct to those of *Aedes*, *Anopheles* and *Culex* species and comparisons of transcriptomic data have identified key proteins which are notably absent in *Toxorhynchites*, indicating their potential role in blood feeding [77,78]. As the salivary glands are key organs in the transmission of mosquito-borne infections, furthering our understanding of the function of these proteins through comparisons to non-hematophagous mosquitoes will be useful in informing new approaches to disease control, such as the development of resistant transgenic vector species. Further to this, a histological study by Pascini and colleagues investigated the distribution and developmental differences of the fat body in *Tx. theobali* [79]. This information adds to our understanding of their physiology and the involvement of the fat body in metabolic activities in *Toxorhynchites* species, which may be used to aid mass rearing programs.

## 5. Olfaction

Mosquito behavior is largely driven by the detection of odorants signaling the location of animal or plant hosts, as well as suitable oviposition sites [71,80,81,82,83]. Despite their highly divergent biology, recent studies have indicated that *Tx. amboinensis* and *Ae. aegypti* share a number of chemosensory and olfactory genes [84], including functional orthologs of odorant receptors, such as indolergic receptors, OR2 and OR10 [85], (R)-1-octen-3-ol receptor, OR8 [86], and sulcatone receptor, OR4 [87]. Sulcatone has previously been suggested to be involved in human host sensing through OR4 in *Ae. aegypti* [88]. However, TambOR4 function in *Tx*. *amboinensis* suggests that it may also have a role in the detection of plant-based sulcatone compounds. Similarly, indole and skatole are considered to be oviposition odorants for multiple species, including *Ae. aegypti* [89], *An. gambiae* [90] and *Cx. quinquefasciatus* [91], as well as having roles in animal-host [92] and plant-host [93] attraction. TaOR2 and TaOR10 have been shown to be highly conserved to those present in their hematophagous counterparts, demonstrating that indole and skatole play multiple roles across a range of mosquito families. Information gathered about olfactory and chemosensory cues in non-blood feeding mosquitoes will provide insights into the signaling pathways involved in blood feeding behavior and help inform the development of improved vector repellent compounds or those that may influence oviposition site selection. 

## 6. Interactions with Viruses

*Toxorhynchites* are considered suitable for release to control medically important vector species due to their nectarivorous nature and lack of blood feeding behavior. As a result, they are not considered to increase the risk of mosquito-borne disease transmission. However, in nature, they may come into contact with arboviruses through ingestion of vertically infected larvae [94], although it is as yet unknown if these could be transstadially transmitted into the adult life stage. Several species have been shown to be susceptible to infection by key arboviruses following intrathoracic inoculation of adults. In particular, flaviviruses, such as all four DENV serotypes, ZIKV, YFV, St. Louis encephalitis virus (SLEV) and Japanese encephalitis virus (JEV), can be effectively propagated in vivo in *Tx. amboinensis*, *Tx. splendens*, *Tx* *rutilus* and *Tx. brevipalpis* [95,96,97,98]. Furthermore, it has been noted that DENV-2 and JEV disseminated to the salivary glands of *Tx. splendens*, which were susceptible to infection [98]. *Tx. amboinensis* have also demonstrated the capacity to replicate other arboviruses in vivo [99], including alphaviruses (Ross River virus [100], CHIKV and Venezuelan encephalitis virus [101]) and bunyaviruses (La Crosse, Keystone and San Angelo viruses [95]), as well as displaying susceptibility to rhabdoviruses (vesicular stomatitis virus [102]) and the true insect virus, Nodamura virus [103]. Due to the success of propagating arboviruses in *Toxorhynchites* in vivo¸ a number of in vitro derived cell lines were established from *Tx. amboinensis* which proved to be as efficient for viral replication. These include TRA-171 (larvae), TA-9 (larvae), TRA-284 (larvae) and TA-42 (eggs) derived cell lines [104,105,106,107,108,109,110,111,112,113,114].

In many cases, viruses were able to replicate in *Tx. amboinensis* both in vivo and in their derived cell lines as well as, or better than, they did in vector species or mammalian systems [95,97,99,101,107,108,109,115]. As a result, they have been selected as the system of choice for the detection of dengue during the manufacture of vaccine candidates [116,117] in addition to having a role in the surveillance of ongoing viral outbreaks through their use as a sensitive insect bioassay, permitting recovery of clinical samples [118,119,120]. Previous work has also investigated their immune responses to infection. Similar to what has been observed in vector species, the *Tx. amboinensis*- derived cell line, TRA-171, demonstrated an active antiviral RNA inference pathway which produced 21 nt viral-derived small RNAs in response to infection with Semliki Forest virus (SFV) [121], while cell death was evident following DENV infection of TRA-171 cells [106,115] and *Tx. splendens* larvae [122]. 

Furthermore, *Toxorhynchites* are also known to be infected by insect specific viruses [121]. These include densoviruses which have been detected both in vivo [123] and in vitro [124]. As this is a comparatively new area of research, there are still a number of unknowns that need to be investigated. For example, understanding how these viruses interact with their host, in particular, the host’s antiviral immune responses, will be a key area of study to establish their potential impact on pathogen transmission and vector competence in medically relevant species. *Toxorhynchites* may provide a useful and safer in vivo system to answer some of these outstanding questions. 

## 7. Interactions with Bacteria

Studies investigating the survival of larvae in changing water quality suggested variations between the tolerances of different species. *Tx. rutilus septentrionalis* larvae died in sewage-contaminated water [46], while laboratory tests demonstrated that *Tx. brevipalpis* is susceptible to bacterial infections [47].

Interestingly, *Ae. aegypti* preferentially oviposited in containers where *Toxorhynchites* were located due to the presence of strong bacterial cues [125]. Findings suggest that despite the danger of predation on their offspring, *Ae. aegypti* choose aquatic habitats where bacterial food for larvae is plentiful. The feeding activities or killing behavior of *Toxorhynchites* can directly or indirectly increase bacterial abundance in oviposition sites through decreasing larval predation on bacteria in that habitat, as well as adding victim body parts which act as substrates for bacteria to feed on. This feature provides further support for the use of *Toxorhynchites* in the control of *Aedes* species. 

## 8. Control Measures

*Toxorhynchites* have periodically been suggested throughout the 20th century as a potential alternative method for the control of vector species with numerous report findings in their favor (Table 1), while others showed less promising results [68,126,127,128,129,130]. Successful control of disease transmission, in particular by diurnal species, such as *Ae. aegypti* and *Ae. albopictus*, is challenging on a number of levels, and many traditional chemical control methods are increasingly recognized as being excessively labor intensive, expensive and unsustainable [5,131,132]. Furthermore, the unregulated use of insecticide products has since led to widespread development of resistance [1,2,4]. In the field, *Toxorhynchites* may provide an appealing alternative to the use of chemicals, particularly in domestic water storage containers.

Previous studies have demonstrated the importance of identifying the most suitable species for the control of the target pest species to achieve successful levels of eradication under defined conditions (reviewed in [11,12,13,28]). For example, during laboratory experiments, *Tx. splendens* has shown preferential predation against *Ae. aegypti* larvae compared to *Ae. albopictus* or *An. sinensis* [58] but will feed on *Cx. quinquefasciatus* when no other prey is available [148]. Alternatively, Nyamah and colleagues showed during field trials that *Tx. splendens* will preferentially feed on *Ae. albopictus* over *Cx. fuscocephala* [136]. The prey species involved will have implications for the target oviposition sites, and selection of a suitable predator which utilizes the same sites will be vital for the success of the program [149]. Studies will also be required to investigate the mortality rate of preoviposition adult females to understand their own susceptibility to predators in target areas, as well as determining their ability to overwinter within those environments. It is known that their use may be limited in temperate regions due to the cooler air and water temperatures. Egg laying, larval development and feeding are affected at low temperatures, which will inhibit their use in certain climates [150,151]. Certainly, the implementation of native *Toxorhynchites* species would be preferable where possible to ensure stability within the environment and to minimize the concerns associated with the release of alien species.

**Figure 2:**
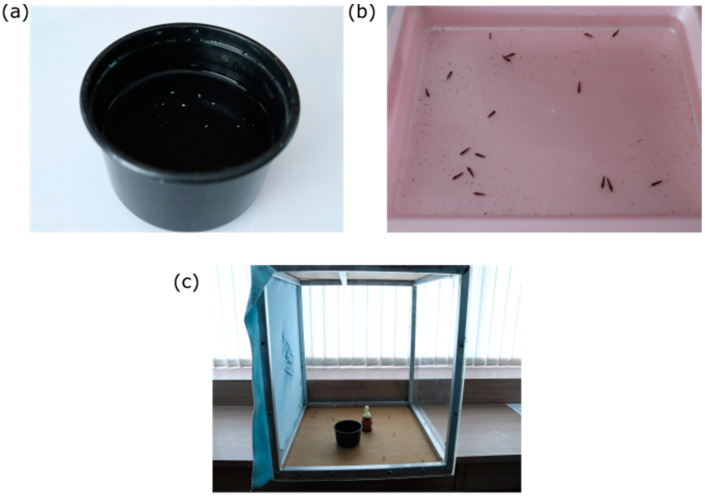
Standard *Toxorhynchites*-rearing containers for (**a**) eggs, (**b**) larvae and (**c**) adults.

Appropriate application of *Toxorhynchites* as a biological control method has been rare due to several restrictions which have limited their practical use. *Toxorhynchites* do not reproduce to sufficient numbers under natural circumstances to keep vector populations under control. Population numbers of *Toxorhynchites* are lower than those of their prey due to the production of significantly fewer eggs, the survival of which declines with the increasing age of the female, as well as an increased larval development time [152,153,154,155]. This is further compounded by the risk of cannibalism between offspring [59]. Therefore, any initiatives require populations to be boosted by locally rearing and regularly releasing additional individuals. Their lack of commercial availability and being hard to rear in large numbers in-house [12,75,156] has made this particularly challenging (Figure 2). Although studies have shown successful results by rearing them individually to prevent cannibalism [53,152] which has overcome some of the difficulties of communal rearing, other hurdles still remain. For instance, large-scale enterprises are costly and difficult to maintain. Encouraging naturally maintained populations by seeding colonies in optimal habitats may also help bolster release numbers and provide a more uniform distribution across the target area. Another factor which influences their efficacy is the density of prey available. In cases of low prey density, *Tx. splendens, Tx. amboinensis* and *Tx. rutilus* demonstrated reduced prey consumption compared to higher prey densities, despite the observation of a higher predatory activity [157,158,159]. It has been suggested that *Toxorhynchites* ‘stock-rear’ their prey, allowing them to conserve limited food supplies which would render them unsuitable as a biological control tool when prey availability is low [160]. Indeed, the risk of disease transmission may increase if this form of predation results in the selected survival of stronger adults [161]. The shape, size and type of container was also a factor which affected the number of prey consumed and may influence their efficacy in different urban and sylvatic environments [158,159]. In particular, this will affect the success of releasing sylvatic, tree-dwelling species into urban environments [68].

It has been recognized that the mere presence of predators can influence prey survival, both through lethal (killing/ consumption) and non-lethal (phenotypic alterations detrimentally affecting prey fitness) means [61]. However, the sole use of predatory mosquitoes rarely results in absolute eradication of target populations and has led to studies investigating their use in combination with other control methods (reviewed in [11,162]). While most chemical insecticides are lethal to both *Toxorhynchites* as well as vector species, appropriately timed releases after treatment can maximize the benefits of both control methods [156]. For instance, *Ae. aegypti* numbers showed a greater decrease following the regular release of *Tx. amboinensis* adult females into a region treated with ultra-low-volume (ULV) malathion pesticide than if either approach was used in isolation [139,163,164]. Further promising strategies have investigated combining *Toxorhynchites* with other biocontrol agents, such as the intracellular bacteria, *Wolbachia* [165], the entomopathogenic fungus, *Metarhizium brunneum* [166], an acetogenin derived from *Annona mucosa* seeds [167] or silver nanoparticles biosynthesized from *Berberis tinctoria* leaf extract [168]. Indeed, as alternative genetic control strategies also become more widespread, it has also been useful to show that *Toxorhynchites* can be successfully employed in combination with the release of transgenic mosquitoes [169]. This is in contrast to data that has indicated that some *Toxorhynchites* species are negatively affected by *Bacillus thuringiensis israelensis* (Bti) and *Bacillus sphaericus* (Bs) biolarvicides [11,170], so this combination is not recommended as part of an integrated control program.

## 9. Conclusions

The need to develop safe, sustainable and environmentally friendly control strategies is key to controlling the spread of mosquito-borne diseases. Previous studies have produced mixed results in favor of the use of *Toxorhynchites* as a biological control agent and there is still much to be done to understand their capacity to sustainably prevent outbreaks of disease through the control of medically important vector populations in field situations. While it is clear that *Toxorhynchites* are not a universal solution, under the correct conditions they have been shown to be very effective. However, more work is required to fully recognize how their use can be optimized, particularly in urban situations. In order to establish a sustainable and effective biological control program, it is important to fully understand the biology of both the control agent as well as the target species. It is vital that the appropriate species of *Toxorhynchites* is identified, as not only will it need to establish itself successfully within the same environment, but it must also have a preference for the consumption of the target population. This can only be achieved through filling in the blanks that exist in our understanding of predator–prey relationships, oviposition cues, nectar sources and population dynamics of *Toxorhynchites*.

Indeed, expanding our knowledge of their biology may provide vital clues into the biology of vector species. Through comparisons with *Toxorhynchites*, we may be better placed to identify key chemo- and odorant receptors involved in blood feeding, as well as to understand the evolution of blood feeding behavior in vector species. Advances in these areas will be beneficial in facilitating the development of targeted repellents or oviposition traps, which will aid in reducing mosquito-borne diseases. Similarly, comparisons between hematophagous and sugar-feeding species will provide insights into the biochemistry and physiology of the major organs involved in pathogen transmission, specifically the salivary glands and the midgut, which will help further our awareness of the adaptations specifically required for blood feeding.

Limiting numbers of natural container breeding sites has been a significant hurdle in the control of mosquito-borne diseases and is an area where deployment of *Toxorhynchites* is likely to be of most use given that it is their nature to actively seek out the habitats commonly missed with other control methods. However, given that captive rearing is challenging and measuring the success of *Toxorhynchites* efficacy in pest species control is both difficult and time consuming, their use is a substantial commitment. Despite these logistical challenges, the data suggest that sufficiently timed releases of *Toxorhynchites* under proper management may provide complete season-long control and make the investment worth the effort, particularly as part of a multimodal, integrated vector management program.

## Figures and Tables

**Table 1:** *Toxorhynchites* species used successfully as biological control agents.

Species (Subgenus)	Geographical Range	Oviposition Preferences	Target Species	Examples of Successful Application
*Tx. splendens* *(Toxorhynchites)*	Asia, Oceania	Artificial containers Tree holes Cut bamboo Leaf axils	*Ae. aegypti* *Ae. albopictus* *Cx. quinquefasciatus*	[133,134,135,136]
*Tx. amboinensis* *(Toxorhynchites)*	Asia, Oceania, North America	Artificial containers Tree holes Cut bamboo Leaf axils	*Ae. polynesiensis* *Ae. aegypti*	[137,138,139,140]
*Tx. rutilus rutilus* *(Lynchiella)*	North America	Artificial containers Tree holes Bromeliads	*Ae. aegypti* *Cx. quinquefasciatus*	[50,141,142]
*Tx. brevipalpis* *(Toxorhynchites)*	Africa	Artificial containers Leaf axils Tree holes	*Ae. aegypti*	[49,143]
*Tx. brevipalpis conradti* *(Toxorhynchites)*	Africa	Artificial containers Leaf axils Tree holes	*Ae. africanus*	[144]
*Tx. moctezuma* *(Lynchiella)*	Central America	Tree holes Cut bamboo	*Ae. aegypti*	[145,146,147]
